# Analytical Investigations and Molecular Dynamics Simulations of 3-Miktoarm Star (3-Arm *μ*-Star) Copolymers *A*_2_*B* and *AB*_2_

**DOI:** 10.3390/ijms27115029

**Published:** 2026-06-02

**Authors:** Pawel Karbowniczek, Zoriana Danel

**Affiliations:** Faculty of Materials Engineering and Physics, Cracow University of Technology, 30-719 Cracow, Poland

**Keywords:** critical phenomena, polymers, molecular dynamics simulations, surface effects

## Abstract

The analytical investigations of 3-miktoarm star (3-arm μ-star) copolymers of type $A_{2}B$ and $AB_{2}$ are performed in the framework of mean-field approximation and Flory–Huggins theory. The total entropy of mixing and the Helmholtz free energy of interaction are calculated for the number $N_{A}$ monomers of type *A* and number $N_{B}$ monomers of type *B*, respectively. The results confirm that the Helmholtz free energy of miktoarm star copolymers differs from that of polymer blends. The temperature dependence of the Helmholtz free energy allowed us to construct a phase diagram of the solution of miktoarm star copolymers, showing regions of stability, instability, and metastability. The analytical results confirm that a miktoarm star copolymer is not merely a mixture of different homo-arm star polymers and are consistent with a previous investigation performed by liquid chromatography under the critical conditions. Moreover, we performed molecular dynamics simulations of a dilute solution of 3-miktoarm star copolymer of type $A_{2}B$ with a certain number of beads (300 + 300 + 200 + 1) and star copolymer of type $AB_{2}$ with number of beads (300 + 200 + 200 + 1), accordingly. The calculations of the radius of gyration and monomer density profiles of the 3-miktoarm star copolymers of type $A_{2}B$ and $AB_{2}$ in confined geometry of two repulsive surfaces (Dirichlet–Dirichlet boundary conditions) and one repulsive and other one attractive surface (Dirichlet–Neumann boundary conditions) by molecular dynamics simulations are performed. The obtained analytical and numerical results indicate that a dilute solution of miktoarm star copolymers can be used in biotechnology and medicine for drug and gene transmission as well as for the production of new functional materials.

## 1. Introduction

The investigation of star polymers, which are a simple example of branched macromolecules with at least three linear chains radiating from a central core, attracts the attention of scientists from both academic and practical points of view due to their unique properties, such as a low viscosity, a high functional group density, and specific core–shell architecture, which enabled several potential applications as thermoplastic elastomers and drug carriers in materials engineering, nanoscience, biology, and medicine (see [1,2] and references therein).

Depending on the chemical compositions of the arms, star polymers can be classified into two categories: (1) star homopolymers, which consist of a symmetrical structure comprising radiating arms with identical chemical composition and similar molecular weight or (2) mikto-arm (or heteroarm) star copolymers, which contain two or more arms with different chemical compositions and can have different molecular weights and different peripheral functionality. As it is known [3], there are several approaches that can be employed for the synthesis of star copolymers: the “core-first” approach, the “arm-first” synthesis, a combination of the “arm-first” and “core-first” approaches, and “grafting onto” a functionalized core via “click” chemistry.

In 1963, Orofino and Wenger [4] used tri(chloromethyl) benzene in combination with anionic polymerization as a linking agent for the first time in order to prepare 3-arm star homopolystyrenes. Some time later, Pennisi and Fetters [5] reported the synthesis of 3-arm asymmetric star homopolymers of homopolystyrenes and polybutadienes. It should be mentioned that, for the first time, synthesis of a 3-arm μ-star copolymer consisting of two polyisoprene and one homopolystyrene arm was presented in [6]. Moreover, in 1992, the synthesis of a 3-arm μ-star terpolymer of homopolystyrenes, polyisoprenes, and polybutadiene was published in [7]. Later in a series of papers [8,9,10,11,12,13,14,15,16], different 3-arm miktoarm star polymers were synthesized by anionic polymerization and selective chlorosilane-based linking chemistry, respectively.

The results of the investigation performed by liquid chromatography under the critical conditions [17] delivered the final proof that a miktoarm star copolymer containing two or more compositionally different arms connected to a single molecule arises. Thus, it was finally confirmed that a miktoarm star copolymer arises, and it is not just a mixture of different homo-arm star polymers.

It should be mentioned that miktoarm star copolymers are a relatively new and unique class of macromolecules, which are interesting due to their intriguing properties that can be tailored by modification of their polymer arms and have found wide applications in medicine, biology, materials engineering, nanotechnology, and nanolithography. There are many papers devoted to these subjects, and below we list some of them. For example, miktoarm star (μ-star) polymers have been extensively investigated in a series of studies as carriers for drug encapsulation [18,19] and anticancer drug delivery applications [20,21,22,23,24,25,26,27] due to their excellent biocompatibility, cell staining performance, and rapid redox responsiveness. Miktoarm star copolymers of type $A_{2}B$ and $AB_{2}$ can easily self-assemble into soft nanoparticles [19]. One of the most common examples of such soft nanoparticles is spherical micelles. Miktoarm star polymers enable the introduction of a different arrangement of hydrophobic/hydrophilic components inside of such micelles. It has been demonstrated that the core–shell structures formed from miktoarm star copolymers upon microphase separation in water yield spherical assemblies with smaller hydrodynamic diameters in comparison to their linear analogs with similar molecular weights and demonstrate higher drug loading efficiencies [22,23].

Moreover, μ-star polymers display exceptional antimicrobial possibilities originating from their tunable biological properties and can be used as antimicrobial agents in order to combat the most common hospital-acquired pathogens and multidrug-resistant Gram-negative bacteria with high therapeutic indices and good selectivity toward pathogens over mammalian cells [28]. It should be mentioned that in the case of using μ-star copolymers as antimicrobial agents, no resistance to bacteria A. baumannii was observed. The extraordinary antimicrobial effectiveness of μ-star polymers was possible due to a multimodal mechanism of bacterial cell death by outer membrane destabilization with the unregulated movement of ions across the cytoplasmic membrane and the induction of the apoptotic-like cell death pathway [28]. Moreover, in [29], an excellent antimicrobial effectiveness of miktoarm star polymers composed of polylysine and glycopolymer toward Gram-positive bacteria was reported.

Miktoarm star polymers can self-assemble into novel and complex morphologies that are inaccessible via the self-assembly of conventional block copolymers. For example, the research presented in [30] was devoted to the fabrication of nanostructured thin films through self-assembly, which has attracted considerable attention as a method for economical generation of the patterns with higher resolution than optical lithography. It should be mentioned that the molecular configuration and structural ordering of miktoarm star polymers in thin films can differ significantly from those in bulk. The reason for such a difference is related to the architectural effects of miktoarm star polymers under confinement, coupled with interfacial interactions at the substrate and surface. Many unique self-assembly phenomena arise when the star structure becomes highly asymmetric, for example, having one A arm and multiple B arms, as is in the case of miktoarm star copolymers of type $A_{2}B$ and $AB_{2}$. This type of arm asymmetry induces significant phase boundary deflection compared to linear polymers due to conformational effects [31,32].

Recently, the influence of solvent quality and substrate-block interactions in thin films of $A_{2}B_{2}$ miktoarm star polymers was investigated by coarse-grained molecular dynamics simulations with a bead-spring model [33]. The results of investigation obtained in [33] indicate that μ-star polymers require a longer time to form a well-ordered cylinder structure than a lamellae structure. In addition, in [33], it was mentioned that, compared to linear block copolymers, μ-star polymers are characterized by much slower self-assembly kinetics with smaller domains. Thus, further investigation of μ-star polymers under confinement is essential to understanding their self-assembly behavior.

The present paper is devoted to an analytical and numerical investigation of the simplest form of 3-arm μ-star copolymers of type $A_{2}B$ and $AB_{2}$ in infinite space and under confinement of two parallel walls with different boundary conditions. Taking into account the mean-field approach and the Flory–Huggins theory, we performed the calculation of the total entropy of mixing and the Helmholtz free energy of mixing for simplified lattice model of 3-arm μ-star copolymers of type $A_{2}B$ and $AB_{2}$ and determine the regions of stability, instability, and metastability of the mixed state for the above-mentioned dilute solution of miktoarm star copolymers. Such an investigation is important because it enables us to obtain the phase diagram of miktoarm star copolymers. Moreover, taking into account the bead–spring model, we performed molecular dynamic calculations of the monomer density profiles for a dilute solutions of 3-arm μ-star copolymers of type $A_{2}B$ and $AB_{2}$ in confined geometries like a slit of two parallel walls with different boundary conditions.

## 2. Results and Discussion

### 2.1. Analytical Results for 3-Miktoarm Star (3-Arm μ-Star) Copolymers $A_{2}B$ and $AB_{2}$

**Figure 1:**
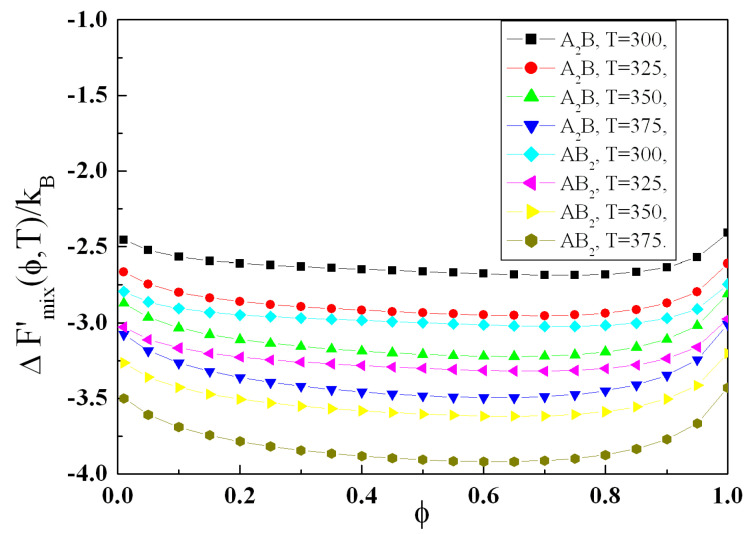
The Helmholtz free energy for hypothetical 3-miktoarm star copolymer $A_{2}B$ as function of ϕ at $\phi_{C}=0.0012$ and for star copolymer $AB_{2}$ as a function of ϕ at $\phi_{C}=0.0014$ for temperatures *T* = 300 K, 325 K, 350 K, 375 K with the Flory interaction parameters $\chi_{1}$ = (2.5 K)/T, $\chi_{2}$ = (3 K)/T, $\chi_{3}$ = (3.5 K)/T.

**Figure 2:**
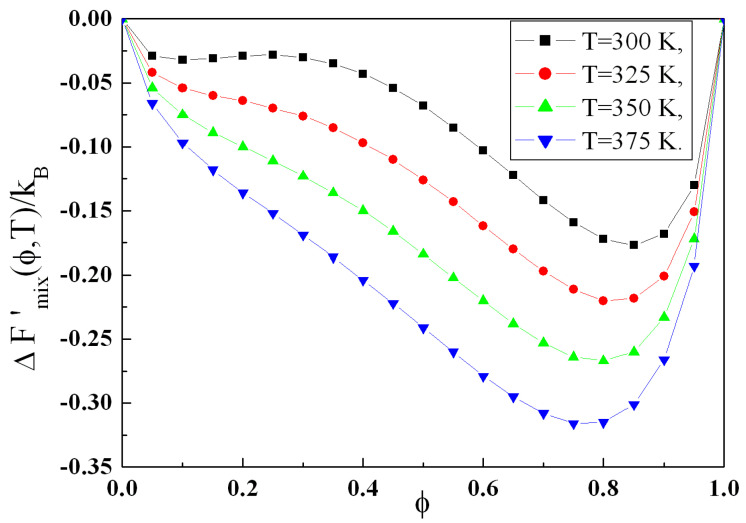
The Helmholtz free energy for hypothetical polymer blends as a function of ϕ at temperatures *T* = 300 K, 325 K, 350 K, 375 K with the Flory interaction parameter χ = (2.5 K)/T obtained according to [34,35].

The results of calculations for the Helmholtz free energy $\Delta F_{mix}^{\prime }\left(\phi ,T\right)/k_{B}$ for the 3-arm μ-star copolymer of type $A_{2}B$ as a function of the volume fraction $\phi_{A}^{\prime }=\phi$ at $\phi_{C}=0.0012$ and $\phi_{C}=0.0014$ for star copolymer $AB_{2}$ for temperatures *T* = 300 K, 325 K, 350 K, 375 K are presented in Figure 1. In Figure 2, we present, for comparison, the results for the Helmholtz free energy of polymer blends with the number $N_{A}$ monomers of type *A* and number $N_{B}$ monomers of type *B* according to the results analyzed in [34].

As it is possible to see from Figure 1, the results for the 3-arm μ-star copolymer of type $A_{2}B$ with number $N_{A}=300$ monomers of type *A* and number $N_{B}=200$ monomers of type *B* are different from the results for polymer blends on Figure 2, with the respective number of monomers of type *A* and *B*.

The results finally confirm that the Helmholtz free energy of 3-miktoarm star copolymers differs from the results for the Helmholtz free energy of polymer blends, as shown in Figure 1 and Figure 2. Thus the obtained analytical results confirm that a 3-miktoarm star copolymer it is not just a mixture of different homo-arm star polymers and are in agreement with the investigation performed using liquid chromatography under the critical conditions [17] as mentioned above.

**Figure 3:**
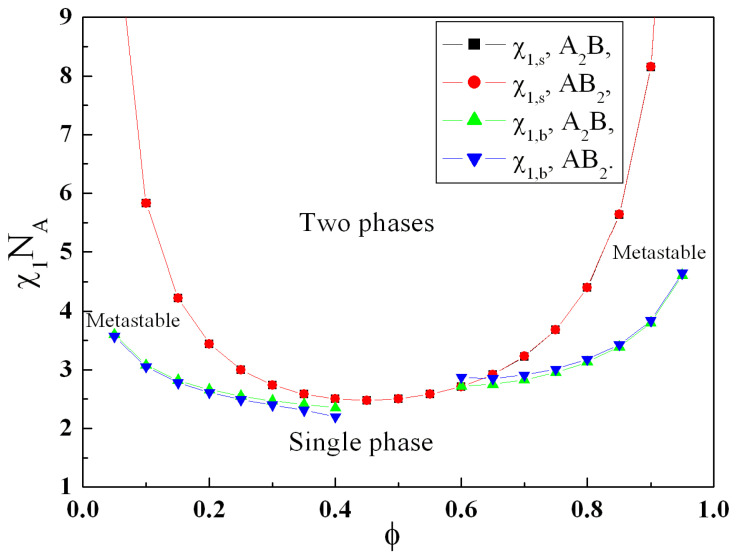
Phase diagram of μ-star copolymers $A_{2}B$ at $\phi_{C}=0.0012$ and $AB_{2}$ at $\phi_{C}=0.0014$. The dependence of $\chi_{1}N_{A}$ from composition ϕ.

The dependence of binodal $\chi_{1,b}$ (see Equation (17) from composition ϕ for μ-star copolymers $A_{2}B$ and $AB_{2}$ is presented on Figure 3. It should be mentioned that the binodal coincides with the coexistence curve.

For the second derivative of the Helmholtz free energy (see Equation(15)) of 3-arm μ-star copolymer, we obtain

(1) $$\frac{\partial^{2}\Delta F_{mix}^{\prime }}{\partial \phi^{2}}=k_{B}T\left(\frac{1}{N_{A}\phi }+\frac{1}{N_{B}\left(1-\phi -\phi_{C}\right)}-2\chi_{1}\right).$$

It should be mentioned that the second derivative of the Helmholtz free energy (see Equation (1)) of 3-arm μ-star copolymers $A_{2}B$ with $\phi_{C}=0.0012$ and $AB_{2}$ with $\phi_{C}=0.0014$ is positive in the whole region of composition 0≤ϕ≤1. The inflection points in $\Delta F_{mix}^{\prime }\left(\phi ,T\right)$ can be found by equating the second derivative of the free energy to zero: $\frac{\partial^{2}\Delta F_{mix}^{\prime }}{\partial \phi^{2}}=0$. The curve corresponding to the inflection point is the boundary between unstable and metastable regions and is named the spinodal, which in our case is

(2) $$\chi_{1,s}=\frac{1}{2}\left(\frac{1}{N_{A}\phi }+\frac{1}{N_{B}\left(1-\phi -\phi_{c}\right)}\right).$$

The lowest point on the spinodal curve corresponds to the critical point and can be obtained by equating the first derivative of the spinodal to zero

(3) $$\frac{\partial \chi_{1,s}}{\partial \phi }=\frac{1}{2}\left(-\frac{1}{N_{A}\phi^{2}}+\frac{1}{N_{B}{\left(1-\phi -\phi_{C}\right)}^{2}}\right)=0.$$

The solving of Equation (3) gives us the critical composition

(4) $$\phi_{cr}=\frac{\left(1-\phi_{C}\right)\sqrt{N_{B}}}{\sqrt{N_{A}}+\sqrt{N_{B}}}.$$

After substituting Equation (4) into Equation (2), we can obtain critical interaction parameter $\chi_{1,cr}\approx 2.4777$ for μ-star copolymer $A_{2}B$ and $\chi_{1,cr}\approx 2.4782$ for μ-star copolymer $AB_{2}$. The dependence of the spinodal $\chi_{1,s}$ from composition ϕ is presented in Figure 3.

The spinodal and binodal curves meet at the critical point, as shown in Figure 3. For interaction parameter $\chi_{1}<\chi_{1,cr}$ the homogeneoas mixture is stable at any composition. For the case $\chi_{1}>\chi_{1,cr}$ there is a miscibility gap between the two branches of the binodal. It should be mentioned that for any composition within a miscibility gap, the equilibrium state consists of two phases. The points on the phase diagram between the spinodal curve and binodal curve correspond to metastable mixtures (see Figure 3). As shown in Figure 3, the phase diagram for miktoarm star copolymers differs from that for polymer blends (see [34]).

### 2.2. Numerical Results for Monomer Density Profiles of 3-Arm μ-Star Copolymers $A_{2}B$ and $AB_{2}$

To solve the equations of motion we used a program written in C++, that implements the Verlet integration scheme with a time step Δt=0.005. Simulations were performed in the NVT ensemble; therefore, we used a thermostat with constant temperature T=1 applied every selected number of time steps (200).

First, in order to determine the radius of gyration of the star polymer and its copolymer counterparts, we performed 20 simulations over $11\times 10^{3}$ time steps ($10^{3}$ for equilibration and $10^{4}$ for data acquisition). We have obtained $R_{g}=29.7(12)$ for homopolymer star with three arms, which is in agreement with our previous result $R_{g}=30.69$ obtained in [36], and then we calculated the radius of gyration $R_{g}=29.82(72)$ for 3-arm μ-star copolymer $A_{2}B$ and $R_{g}=29.33(98)$ for 3-arm μ-star copolymer $AB_{2}$. Therefore, as seen from these results, there are no statistically significant differences in the radius of gyration of copolymers with the same length of arms. Then, we performed simulations with different boundary conditions: two repulsive (Dirichlet–Dirichlet) and repulsive–attractive (Dirichlet–Neumann) walls. In both cases, we analyzed the situations where the distance between walls is equal to $2R_{g}$ and $R_{g}/2$. In order to present the results, we normalized the distance between the walls to 1 and each density profile to $\int_{0}^{1}\rho \left(z\right)dz=\frac{R_{g}}{L}$.

**Figure 4:**
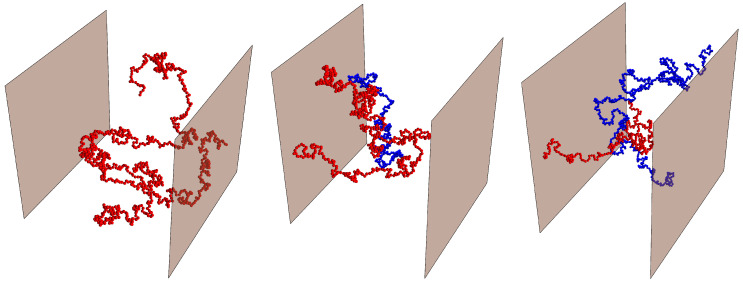
Exemplary configurations of star polymer with three arms and 3-arm μ-star copolymers $A_{2}B$ and $AB_{2}$ for the case of two repulsive walls at the distance $L=2R_{g}$. Red color of monomers corresponds to the normal arm with ϵ=1 and blue color to copolymer arms with ϵ=4.

**Figure 5:**
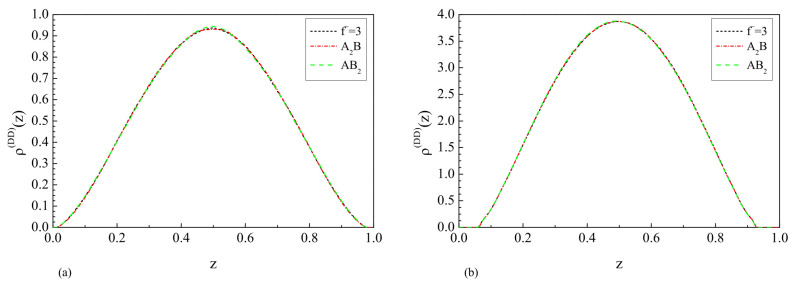
Monomer density profiles ρ(z) of star-shaped polymer with three arms and 3-arm μ-star copolymers $A_{2}B$ and $AB_{2}$ between two repulsive walls. The separation of the walls is equal to (**a**) L=2Rg and (**b**) $L=R_{g}/2$.

**Figure 6:**
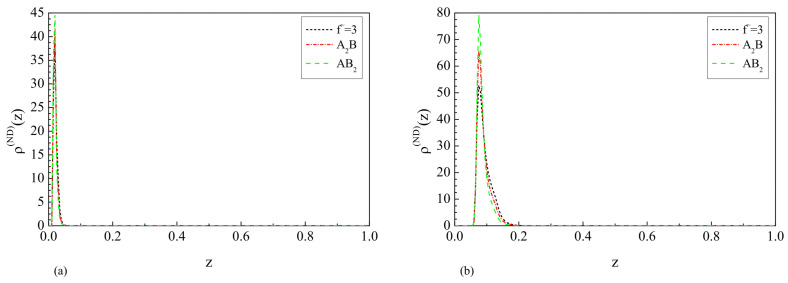
Monomer density profiles ρ(z) of star-shaped polymer and 3-arm μ-star copolymers $A_{2}B$ and $AB_{2}$ between one attractive and one repulsive wall. The separation of the walls is equal to (**a**) L=2Rg and (**b**) $L=R_{g}/2$.

Exemplary configurations of star polymer with three arms and copolymers between two walls with separation $L=2R_{g}$ for the Dirichlet–Dirichlet boundary conditions are presented in Figure 4. Monomer density profiles, presented in Figure 5 and Figure 6, suggest that there is no difference between star homopolymers and copolymers $A_{2}B$ and $AB_{2}$ for two repulsive walls in the case where we have the copolymers with the same length of arms. However, there is a significant difference in the adsorption of polymers for the case of one attractive wall, where copolymers adsorb better than the usual star-shaped polymers because of the stronger interaction with the wall. The difference is caused by the shape of the wall-particle potential. The repulsive part of the Lennard–Jones 9-3 potential is very strongly inclined, and therefore, the change in its effect for copolymers is almost imperceptible. However, for attractive interaction, the potential has a minimum that is greater for copolymers, and this causes an increased absorption to the wall.

**Figure 7:**
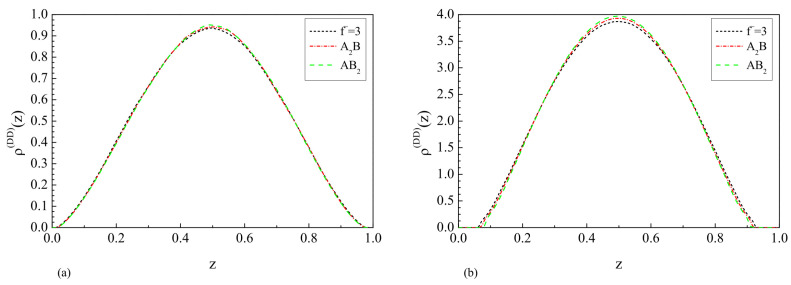
Monomer density profiles ρ(z) of star-shaped polymer with 3 arms and 3-arm μ-star copolymers $A_{2}B$ and $AB_{2}$ with different length of arms between two repulsive walls. The separation of the walls is equal to (**a**) L=2Rg and (**b**) $L=R_{g}/2$.

**Figure 8:**
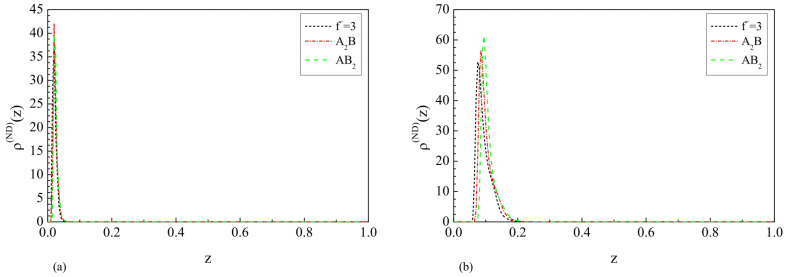
Monomer density profiles ρ(z) of star-shaped polymer with 3 arms and 3-arm μ-star copolymers $A_{2}B$ and $AB_{2}$ with different length of arms between one attractive and one repulsive wall. The separation of the walls is equal to (**a**) L=2Rg and (**b**) $L=R_{g}/2$.

Moreover, we analyzed the influence of the lengths of μ-star copolymer arms on the adsorption properties. Arms interacting with the Lennard–Jones potential with parameter ϵ=4 were built of 200 monomers, giving a total number of 801 monomers (300+300+200+1) for $A_{2}B$ copolymer and 701 monomers (300+200+200+1) for $AB_{2}$ copolymer. First, we have calculated $R_{g}=27.62(78)$ for $A_{2}B$ copolymer and $R_{g}=25.21(62)$ for $AB_{2}$ copolymer. Then, we simulated the above mentioned copolymers in a slit with Dirichlet–Dirichlet and Dirichlet–Neumann boundary conditions. For such polymers, monomer density profiles are presented in Figure 7 and Figure 8. Like in the previous case, there is a significant difference in polymer adsorption for the case of one attractive and one repulsive wall (see Figure 8). However, the reduced length of copolymer arms that interact with a stronger potential also caused the differences for two repulsive walls (see Figure 7). In such a case, copolymers $A_{2}B$ and $AB_{2}$ are more localized in the center of a slit than a homopolymer star with $\tilde{f}=3$.

## 3. Materials and Methods

### 3.1. Analytical Investigation of 3-Miktoarm Star (3-Arm μ-Star) Copolymers $A_{2}B$ and $AB_{2}$

We apply a mean-field approach for the description of 3-arm μ-star copolymers of type $A_{2}B$, where we assume that we have two arms of type *A* and one arm of type *B*. Moreover, we performed an investigation of the μ-star copolymer of type $AB_{2}$, where we assumed that we have one arm of type *A* and two arms of type *B*. We assume that our copolymers can be modeled as a sequence of repeating units of two types, *A* and *B*, on the periodic lattice, and that the lattice sites are of the order of monomer sizes, but do not necessarily correspond exactly to the chemical monomers. We do not consider charged systems such as polyelectrolytes. The mean-field approach for polymer blends with two different macromolecular chains with length $N_{A}>>1$ and $N_{B}>>1$ [34] gives the possibility to calculate the total entropy of mixing per lattice site:

(5) $$\Delta {\tilde{S}}_{mix}=\frac{\Delta S_{mix}}{n}=-k_{B}\left(\frac{\phi_{A}}{N_{A}}ln\phi_{A}+\frac{\phi_{B}}{N_{B}}ln\phi_{B}\right),$$

where $\phi_{A}$ and $\phi_{B}$ are the volume fraction of the two components in polymer blends:

(6) $$\begin{array}{c} \phi_{A}=\frac{V_{A}}{V_{A}+V_{B}},\\ \phi_{B}=\frac{V_{B}}{V_{A}+V_{B}}, \end{array}$$

and $V_{A}=N_{A}\sigma$ is the molecular volume of a molecule of species *A*, $V_{B}=N_{B}\sigma$ is the molecular volume of a molecule of species *B*, and σ is the lattice site volume. It should be mentioned that $N_{A}$ and $N_{B}$ are the numbers of lattice sites occupied by molecules of type *A* and type *B*, respectively. Taking into account the mean-field approach, we can obtain the total entropy of mixing per lattice site for the 3-arm μ-star copolymer:

(7) $$\Delta {\tilde{S}}_{mix}^{\prime }=\frac{\Delta S_{mix}^{\prime }}{n^{\prime }}=-k_{B}\left(\frac{\phi_{A}^{\prime }}{N_{A}}ln\phi_{A}^{\prime }+\frac{\phi_{B}^{\prime }}{N_{B}}ln\phi_{B}^{\prime }+\frac{\phi_{C}^{\prime }}{N_{C}}ln\phi_{C}^{\prime }\right),$$

where $\phi_{A}^{\prime }$, $\phi_{B}^{\prime }$, and $\phi_{C}^{\prime }$ are the volume fraction of the A,B,C components in the 3-arm μ-star copolymer. For the case of a 3-arm μ-star copolymer with configuration $A_{2}B$, we have

(8) $$\begin{array}{c} \phi_{A}^{\prime }=\frac{2N_{A}}{2N_{A}+N_{B}+N_{C}},\\ \phi_{B}^{\prime }=\frac{N_{B}}{2N_{A}+N_{B}+N_{C}},\\ \phi_{C}^{\prime }=\frac{1}{2N_{A}+N_{B}+N_{C}}, \end{array}$$

where we assume that we have one monomer $N_{C}=1$ of type *C* in the center of μ-star polymer. For the case of 3-arm μ-star copolymer $AB_{2}$, we have, respectively,

(9) $$\begin{array}{c} \phi_{A}^{\prime }=\frac{N_{A}}{N_{A}+2N_{B}+N_{C}},\\ \phi_{B}^{\prime }=\frac{2N_{B}}{N_{A}+2N_{B}+N_{C}},\\ \phi_{C}^{\prime }=\frac{1}{N_{A}+2N_{B}+N_{C}}. \end{array}$$

We can perform calculations for the total entropy of mixing for 3-arm μ-star copolymer $A_{2}B$ in the case where we have two arms of *A* type with $N_{A}=300$ monomers and one arm of *B* type with $N_{B}=200$ monomers, and one monomer in the center, which give us the total number of monomers: N=300+300+200+1. We obtain $\Delta {\tilde{S}}_{mix}^{\prime }=0.0105k_{B}$. It should be mentioned that, for polymer blends, in this case, we have $\Delta {\tilde{S}}_{mix}=0.0025k_{B}$. The respective calculations for 3-arm μ-star copolymer of type $AB_{2}$ with N=300+200+200+1 give us the following result: $\Delta {\tilde{S}}_{mix}^{\prime }=0.012k_{B}$. For polymer blends, in this case, we obtain $\Delta {\tilde{S}}_{mix}=0.0028k_{B}.$ As detailed, the mixing entropy is small for blends and μ-star copolymers, yet always positive, thereby promoting mixing. In addition, the mixing entropy for polymer blends is definitely smaller than the mixing entropy for μ-star polymers. The result in Equation (5) gives the possibility to obtain the free energy of mixing per site for polymer blends [34]:

(10) $$\Delta {\tilde{F}}_{mix}=-T\Delta {\tilde{S}}_{mix}=k_{B}T\left(\frac{\phi_{A}}{N_{A}}ln\phi_{A}+\frac{\phi_{B}}{N_{B}}ln\phi_{B}\right).$$

By analogy, taking into account the result in Equation (7), we can calculate the free energy of mixing per site for 3-arm μ-star copolymers:

(11) $$\Delta {\tilde{F}}_{mix}^{\prime }=-T\Delta {\tilde{S}}_{mix}^{\prime }=k_{B}T\left(\frac{\phi_{A}^{\prime }}{N_{A}}ln\phi_{A}^{\prime }+\frac{\phi_{B}^{\prime }}{N_{B}}ln\phi_{B}^{\prime }+\frac{\phi_{C}^{\prime }}{N_{C}}ln\phi_{C}^{\prime }\right).$$

In the general case, the free energy of mixing can be either negative, which promotes mixing, or positive, in which case mixing is opposed. To calculate the Helmholtz free energy of mixing, we can apply the Flory–Huggins theory [35], a simplified lattice model in which components are mixed at constant volume, including into calculations the energy of interactions between components. Thus, as it was presented in [34,35], the energy change on mixing per lattice site for polymer blends is

(12) $$\Delta {\tilde{U}}_{mix}=k_{B}T\chi \phi_{A}\phi_{B},$$

where $\chi =\frac{z}{2}\left(\frac{2u_{AB}-u_{AA}-u_{BB}}{k_{B}T}\right)$ is the Flory interaction parameter for polymer blends, *z* is the number of nearest neighbors and z=4 for a square lattice, and z=6 for a cubic lattice. The average pairwise interaction of a monomer of type *A* with one of its neighboring monomers is just a volume fraction weighted sum of interaction energies between monomers: $U_{A}=\phi_{A}u_{AA}+\phi_{B}u_{AB}$. For the monomer of type *B* the average pairwise interaction with one of its neighbors is $U_{B}=\phi_{A}u_{AB}+\phi_{B}u_{BB}$. Taking into account the Flory–Huggins theory [35], for the energy change per lattice site of 3-arm μ-star copolymer we obtain

(13) $$\Delta {\tilde{U}}_{mix}^{\prime }=k_{B}T\left(\chi_{1}\phi \left(1-\phi -\phi_{c}\right)+\chi_{2}\phi_{c}\left(1-\phi -\phi_{c}\right)+\chi_{3}\phi \phi_{C}\right),$$

where we introduced the following interaction parameters $\chi_{1},\chi_{2},\chi_{3}$:

(14) $$\begin{array}{c} \chi_{1}=\frac{z}{2}\left(\frac{2u_{AB}-u_{AA}-u_{BB}}{k_{B}T}\right),\\ \chi_{2}=\frac{z}{2}\left(\frac{2u_{BC}-u_{BB}}{k_{B}T}\right),\\ \chi_{3}=\frac{z}{2}\left(\frac{2u_{AC}-u_{AA}}{k_{B}T}\right). \end{array}$$

Here we took into account that, in the case of 3-miktoarm star copolymers, the average pairwise interaction of a monomer of type *A* with one of its neighboring monomers is $U_{A}^{\prime }=\phi_{A}^{\prime }u_{AA}+\phi_{B}^{\prime }u_{AB}+\phi_{C}^{\prime }u_{AC}$. For the monomer of type *B* in the case of 3-arm μ-star copolymers, the average pairwise interaction with one of its neighbors is $U_{B}^{\prime }=\phi_{A}^{\prime }u_{AB}+\phi_{B}^{\prime }u_{BB}+\phi_{C}^{\prime }u_{BC}$, and for the central monomer of type *C*, we have $U_{C}^{\prime }=\phi_{A}^{\prime }u_{AC}+\phi_{B}^{\prime }u_{BC}.$ Taking into account the above result in Equation (13), we can obtain the result for the Helmholtz free energy of mixing per lattice cite for the 3-arm μ-star copolymer:

(15) $$\begin{array}{ccc} \Delta F_{mix}^{\prime }=\Delta {\tilde{U}}_{mix}^{\prime }-T\Delta {\tilde{S}}_{mix}^{\prime } & = & k_{B}T(\frac{\phi }{N_{A}}ln\phi +\frac{\left(1-\phi -\phi_{C}\right)}{N_{B}}ln\left(1-\phi -\phi_{C}\right)+\\ +\frac{\phi_{C}}{N_{C}}ln\phi_{C}+\chi_{1}\phi \left(1-\phi -\phi_{C}\right) & + & \chi_{2}\phi_{C}\left(1-\phi -\phi_{C}\right)+\chi_{3}\phi \phi_{C}), \end{array}$$

where we introduced the following notations: $\phi_{A}^{\prime }=\phi$, $\phi_{B}^{\prime }=1-\phi_{A}^{\prime }-\phi_{C}^{\prime }=1-\phi -\phi_{C}$, and $\phi_{C}^{\prime }=\phi_{C}$. As it is possible to see from Equation (15), the first three terms in this equation have entropic origin, and in accordance with it, they always promote mixing. The last three terms in Equation (15) have energetic origin and can be positive, zero or negative depending on the sign of the respective interaction parameters $\chi_{1}$, $\chi_{2}$, $\chi_{3}$.

One of the major assumptions of the Flory–Huggins theory is that there is no volume change during mixing, and the monomers of both species can fit on the lattice sites of the same lattice. As is known, in most real polymer blends, the volume per monomer changes after mixing. Additionally, some monomers may pack together better with certain other monomers. In the general case, all deviations from the lattice model are collected into the interaction parameters $\chi_{1}$, $\chi_{2}$ and $\chi_{3}$, which display non-trivial dependences on composition, temperature, and chain length. We can assume that in the case of 3-arm μ-star copolymers the temperature dependences of the interaction parameters can be written as the sum of two terms: $\chi_{1}=\alpha_{1}+\beta_{1}/T$, $\chi_{2}=\alpha_{2}+\beta_{2}/T$ and $\chi_{3}=\alpha_{3}+\beta_{3}/T$, where $\alpha_{i}$ is “entropic part” and $\beta_{i}/T$ with i=1,2,3 is “entalpic part” of interaction parameters. The temperature dependence of the Helmholtz free energy (see Equation (11)) allows us to construct a phase diagram that summarizes the phase behavior of solutions of miktoarm star polymers, showing regions of stability, instability, and metastability. In order to obtain the interaction parameter corresponding to the phase boundary, which is named binodal, we need to calculate the first derivative of the Helmoltz free energy with respect to composition and solve the following Equation

(16) $$\frac{\partial \Delta F_{mix}^{\prime }}{\partial \phi }=0.$$

Taking into account the result for the Helmholtz free energy in Equation (15) and solving Equation (16) for the binodal, we obtain

(17) $$\chi_{1,b}=\frac{1}{\left(2\phi +\phi_{C}-1\right)}\left(\frac{1}{N_{A}}-\frac{1}{N_{B}}+\frac{\ln\phi }{N_{A}}-\frac{\ln(1-\phi -\phi_{C})}{N_{B}}-\phi_{C}\chi_{2}+\phi_{C}\chi_{3}\right).$$

The local stability of such a solution of miktoarm star polymers is determined by the sign of the second derivative of the Helmholtz free energy (see Equation (11)) with respect to composition ϕ

(18) $$\begin{array}{ccc} \frac{\partial^{2}\Delta F_{mix}^{\prime }}{\partial \phi^{2}} & < & 0,-unstable,\\ \frac{\partial^{2}\Delta F_{mix}^{\prime }}{\partial \phi^{2}} & > & 0,-locally\;stable. \end{array}$$

### 3.2. Molecular Dynamics Simulations of 3-Arm μ-Star Copolymers $A_{2}B$ and $AB_{2}$

To confirm our theoretical results, we performed molecular dynamics simulations of star-shaped copolymers with 3 arms. At the beginning, we investigated the case where polymers were built from 1 central monomer plus 3 arms, each consisting of 300 monomers, for a total number of 901 monomers.

The interaction between neighboring monomers in the polymer chain was modeled using FENE (finite extensible nonlinear elastic potential) [37]:

(19) $$U_{FENE}\left(r\right)=\left\{\begin{array}{cc} -\frac{1}{2}kR_{0}^{2}\ln\left[1-{\left(\frac{r}{R_{0}}\right)}^{2}\right] & \mathrm{if}\;r<R_{0},\\ +\infty & \mathrm{if}\;r\geq R_{0}, \end{array}\right.$$

where we assumed k=30 and $R_{0}=3/2$ and the WCA (Weeks–Chandler–Andersen) potential [38]:

(20) $$U_{WCA}\left(r\right)=\left\{\begin{array}{cc} -\epsilon & \mathrm{if}\;r<2^{1/6}\sigma ,\\ 4\epsilon \left[{\left(\frac{\sigma }{r}\right)}^{12}-{\left(\frac{\sigma }{r}\right)}^{6}\right] & \mathrm{if}\;r\geq 2^{1/6}\sigma . \end{array}\right.$$

Monomers, which were not neighbors, interacted via the 12-6 Lennard Jones potential [39]:

(21) $$U_{12-6}\left(r\right)=4\epsilon \left[{\left(\frac{\sigma }{r}\right)}^{12}-{\left(\frac{\sigma }{r}\right)}^{6}\right].$$

The interaction with the walls was given by the 9-3 Lennard–Jones potential [40]:

(22) $$U_{9-3}\left(r\right)=\frac{3\sqrt{3}}{2}\epsilon \left[{\left(\frac{\sigma }{r}\right)}^{9}-{\left(\frac{\sigma }{r}\right)}^{3}\right].$$

This potential was used with a cut-off, which determined the type of polymer–wall interaction. For $R_{cut-off}=3^{1/6}$, the wall was repulsive, and for $R_{cut-off}=10$ attractive.

For the standard star-shaped polymer, we assumed ϵ=1 and σ=1. However, in order to construct 3-arm μ-star copolymer, at least one arm has to interact differently. We assumed that the monomers of such arms have to interact with the greater strength represented by the different depth of the potential ϵ=4. We also assumed that all monomers have the same size σ=1. By applying this, we also had to modify interactions with other arms and walls. In this case, we used well established Lorentz–Berthelot mixing rules [41,42] for the monomer radii

(23) $$\sigma_{ij}=\frac{\sigma_{ii}+\sigma_{jj}}{2}$$

and diameters

(24) $$\epsilon_{ij}=\sqrt{\epsilon_{ii}\epsilon_{jj}}$$

for the potential depth.

## 4. Conclusions

The analytical investigation of 3-miktoarm star ( 3-arm μ-star) copolymers of type $A_{2}B$ and $AB_{2}$ is performed in the framework of the mean-field approach and Flory–Huggins theory. The total entropy of mixing and the Helmholtz free energy of interaction of 3-miktoarm star copolymers were calculated. The obtained results confirm that the Helmholtz free energy and phase diagram for miktoarm star copolymers differ from those for polymer blends, as shown in Figure 1, Figure 2, Figure 3, respectively. That means that a solution of miktoarm star copolymers is not just a mixture of different homo-arm star polymers, and this result is in agreement with previous investigation performed using liquid chromatography under the critical conditions. Our investigations allow us to determine the regions of stability, instability, and metastability for a dilute solutions of miktoarm star copolymers of type $A_{2}B$ and $AB_{2}$. Moreover, we performed the calculation of the monomer density profiles of 3-miktoarm star copolymers of type $A_{2}B$ and $AB_{2}$ in confined geometry of two repulsive surfaces, which corresponds to Dirichlet–Dirichlet boundary conditions and one repulsive and other one attractive surface, which corresponds to Dirichlet–Neumann boundary conditions by molecular dynamics simulations. We analyzed the influence of the lengths of μ-star copolymer arms on the adsorption properties. Arms interacting with the Lennard–Jones potential with parameter ϵ=4 were built of 200 monomers, and for the arms with 300 monomers, we assumed ϵ=1, giving a total amount of 801 monomers (300+300+200+1) for $A_{2}B$ copolymer and 701 monomers (300+200+200+1) for $AB_{2}$ copolymer, respectively.

We obtained that the radius of gyration for μ-star copolymer $A_{2}B$ is equal to $R_{g}=27.62(78)$ and for μ-star copolymer $AB_{2}$ is $R_{g}=25.21(62)$. These results are smaller than the result for the radius of gyration for a star homopolymer with three arms, which is $R_{g}=29.7(12)$. We determined that there is a significant difference in polymer adsorption for the case of one attractive and other one repulsive wall in the case of 3-miktoarm star copolymers $A_{2}B$ and $AB_{2}$, as shown in Figure 8. However, we found that the reduced length of copolymer arms interacting with a stronger potential also caused differences for two repulsive walls, as shown in Figure 7. The results indicate that in this case, 3-miktoarm star copolymers $A_{2}B$ and $AB_{2}$ are more localized in the center of a slit than a homopolymer star with $\tilde{f}=3$.

Miktoarm star copolymers of type $A_{2}B$ and $AB_{2}$ have been widely used in medicine for drug delivery [43,44,45,46]. As mentioned above, miktoarm star copolymers of type $A_{2}B$ and $AB_{2}$ can easily self-assemble into soft nanoparticles (or micelles) [19] and offer the possibility to introduce a different arrangement of hydrophobic/hydrophilic components inside of micelles. A series of papers [22,23] have demonstrated that the core–shell structures formed from miktoarm star copolymers upon microphase separation in water yield spherical assemblies with smaller hydrodynamic diameters in comparison to their linear analogs with similar molecular weights, and in accordance with this, demonstrate higher drug loading efficiencies. Our obtained numerical results indicate that the radius of gyration for miktoarm star copolymers of type $A_{2}B$ and $AB_{2}$ is smaller than the radius of gyration for homopolymer star [36] with three legs and the radius of gyration for linear polymer chain [47] with the same number of monomers. This result suggests that such miktoarm star copolymers can achieve higher drug-loading efficiencies than linear polymers and homopolymer stars.

Molecular dynamics simulations suggest that the monomer density profiles for 3-arm μ-star copolymers $A_{2}B$ and $AB_{2}$ are bigger near the adsorbing wall than the respective monomer density for a homopolymer star with three arms, as it is possible to see from Figure 6 and Figure 8. This causes a larger number of monomers in the miktoarm star copolymer to be situated near the adsorbing wall, thereby imposing a greater pressure on the wall than in homopolymer star solutions. This assumes a reduction in static friction in such materials, enabling using them for production of new types of MEMS and NEMS. Thus, the obtained analytical and numerical results indicate that a dilute solution of 3-miktoarm star copolymers can find practical application in biotechnology and medicine for drug and gene transmission, as well as for the production of new functional materials.

## Data Availability

The original contributions presented in this study are included in the article. Further inquiries can be directed to the corresponding author.
